# A new genus and tribe of freshwater mussel (Unionidae) from Southeast Asia

**DOI:** 10.1038/s41598-018-28385-y

**Published:** 2018-07-03

**Authors:** Ivan N. Bolotov, John M. Pfeiffer, Ekaterina S. Konopleva, Ilya V. Vikhrev, Alexander V. Kondakov, Olga V. Aksenova, Mikhail Yu. Gofarov, Sakboworn Tumpeesuwan, Than Win

**Affiliations:** 1 Northern Arctic Federal University, Arkhangelsk, Russia; 2 Federal Center for Integrated Arctic Research, Russian Academy of Sciences, Arkhangelsk, Russia; 3 Florida Museum of Natural History, University of Florida, Gainesville, USA; 4 Department of Biology, Faculty of Science, Mahasarakham University, Maha Sarakham, Thailand; 5 Department of Zoology, Hpa-An University, Hpa-An, Kayin State Myanmar

## Abstract

The freshwater mussel genus *Oxynaia* Haas, 1911 is thought to be comprised of two geographically disjunct and morphologically variable species groups but the monophyly of this taxon has yet to be tested in any modern cladistic sense. This generic hypothesis has important systematic and biogeographic implications as *Oxynaia* is the type genus of the currently recognized tribe Oxynaiini (Parreysiinae) and is one of the few genera thought to cross several biogeographically important barriers in Southeast Asia. Morphological and molecular data clearly demonstrate that *Oxynaia* is not monophyletic, and the type species and its allies (*O. jourdyi* group) belong to the Unioninae, and more specifically as members of the genus *Nodularia* Conrad, 1853. Therefore, neither *Oxynaia* **syn. nov**. nor Oxynaiini Starobogatov, 1970 are applicable to the Parreysiinae and in the absence of an available name, *Indochinella* **gen. nov**. and Indochinellini **trib. nov**. are described. Several combinations are proposed as follows: *Indochinella pugio* (Benson, 1862) **gen. et comb. nov**., *Nodularia jourdyi* (Morlet, 1886) **comb. res**., *N. gladiator* (Ancey, 1881) **comb. res**., *N. diespiter* (Mabille, 1887) **comb. res**. and *N. micheloti* (Morlet, 1886) **comb. res**. Finally, we provide an updated freshwater biogeographic division of Southeast Asia.

## Introduction

Integrative taxonomic studies are of substantial practical importance to conservation stakeholders as accurate information on the systematics and distributions of biodiversity forms the foundation of taxon- and habitat-based conservation efforts. The application of basic systematic and phylogenetic research has played a critical role in the conservation of freshwater mussels (Bivalvia: Unionidae), which are among the most threatened groups of animals worldwide^1^. However, the vast majority of recent systematic research has focused on the North American and European fauna, while the comparatively diverse tropical lineages have received disproportionately less attention^2^. Although, several recent systematic efforts focused on Asian lineages have dramatically improved our understanding of the classification, morphological evolution, and biogeography of many tropical freshwater mussel clades^3–9^, many biographically interesting and systematically important taxa remain poorly understood from a phylogenetic perspective. This is particularly true of the genus *Oxynaia*, which has an unusual disjunct geographic distribution in Myanmar and northern Vietnam and is the type genus of the tribe Oxynaiini Starobogatov, 1970.

Whelan *et al*.^10^ recently transferred the tribe Oxynaiini from the subfamily Ambleminae to the Parreysiinae on the basis of recovering *Oxynaia pugio* (Benson, 1862) among the latter subfamily in a molecular phylogeny. Whereas, traditional morphological classifications consistently consider *Oxynaia* a member of the subfamily Unioninae (in its various usages^11–14^). However, these seemingly irreconcilable subfamily-level classifications of *Oxynaia* (Unioninae vs Parreysiinae) have relied primarily on only one of the two geographically disjunct species groups, suggesting that *Oxynaia* may not be monophyletic.

**Figure 1:**
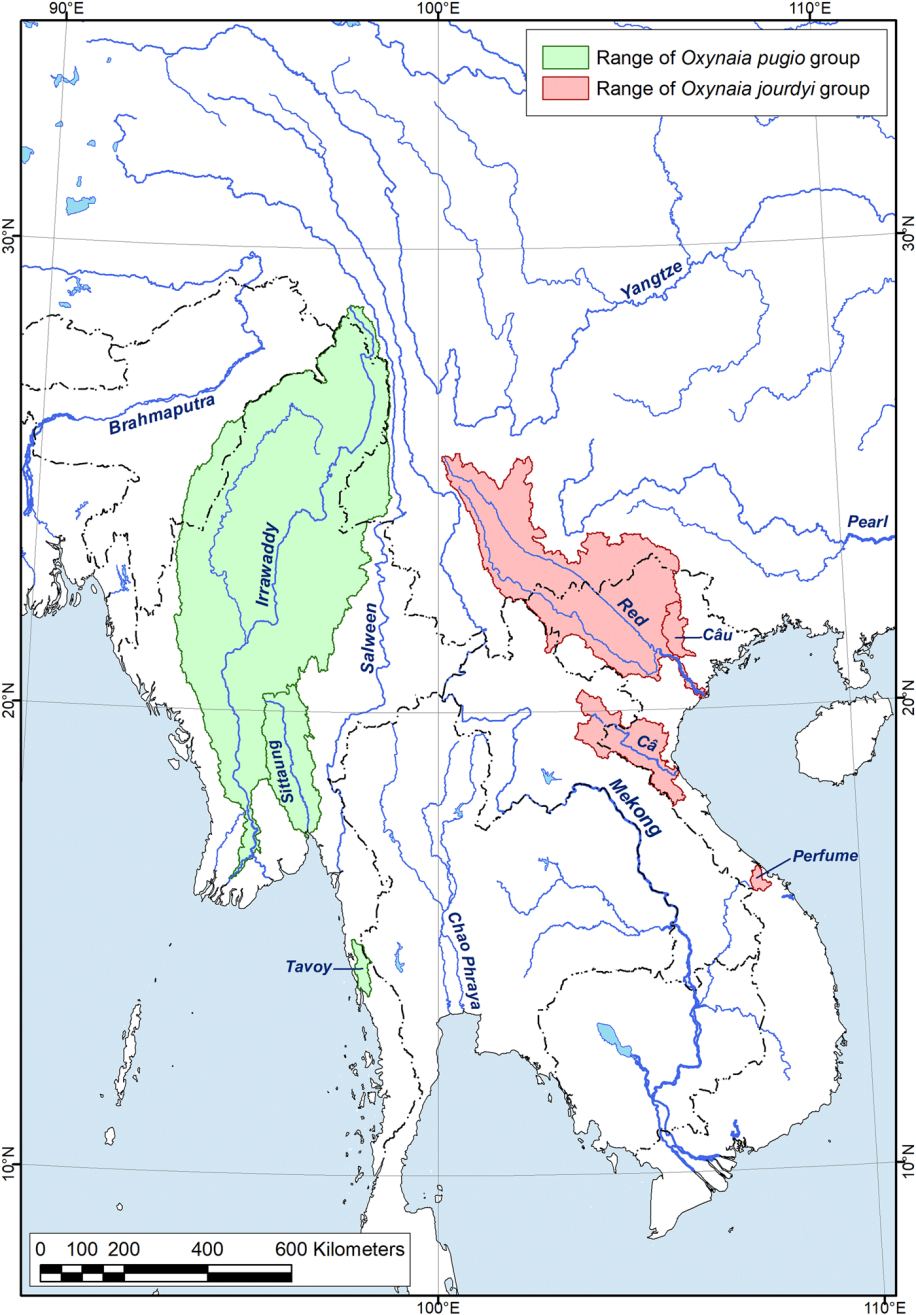
Map of distribution ranges of the two *Oxynaia* species groups in Southeast Asia (see Taxonomic Account for details). The map was created using ESRI ArcGIS 10 software (www.esri.com/arcgis); the topographic base of the map was created with Natural Earth Free Vector and Raster Map Data (www.naturalearthdata.com). (Map: Mikhail Yu. Gofarov).

Species in the genus *Oxynaia* each share the presence of a strongly pointed posterior end (Greek: oxy – ‘sharp’), but can be divided into two distinct geographic species groups, the *O. jourdyi* group and the *O. pugio* group^15^ (Fig. 1). The *Oxynaia jourdyi* group includes four putative species from northern Vietnam: *O. jourdyi* (Morlet, 1886) (generic type), *O. gladiator* (Ancey, 1881), *O. diespiter* (Mabille, 1887), and *O. micheloti* (Morlet, 1886). The *Oxynaia pugio* group contains a single described species (*O. pugio*) from Myanmar^7,10,14^.

Our objective herein is to test the monophyly of the genus *Oxynaia*, evaluate the morphological traits of the resultant suprageneric clades containing *Oxynaia* species, and to make the appropriate taxonomic changes to more accurately reflect our hypotheses of evolutionary history.

## Results

### Phylogenetic analyses

**Figure 2:**
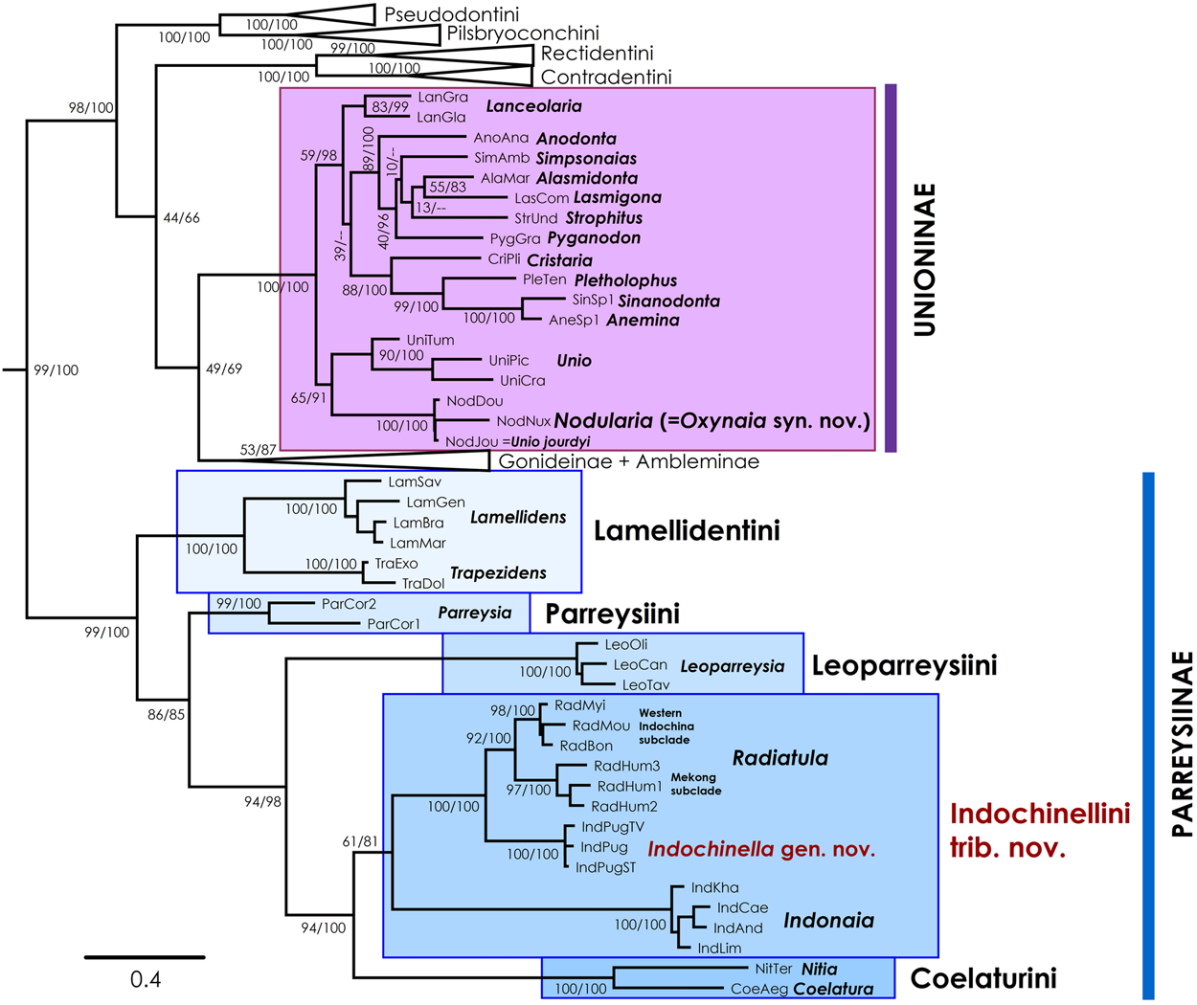
Consensus phylogenetic tree of the Unionidae recovered from ML analysis and obtained for the complete data set of mitochondrial and nuclear sequences (five partitions: three codons of *COI* + 16*S rRNA* + 28*S rRNA*). Black numbers near nodes are bootstrap support values/Bayesian posterior probabilities. The names of taxa under revision are in red. The Rectidentinae, Pseudodontinae, Gonideinae and Ambleminae clades are collapsed. Outgroup taxa are not shown.

Our family-level phylogenetic analyses based on mitochondrial and nuclear markers (five partitions: three codons of *COI* + 16*S rRNA* + 28*S rRNA*) reveals that *Oxynaia* is not monophyletic (Fig. 2). *Oxynaia jourdyi* is well supported as a member of the subfamily Unioninae, whereas *O. pugio* is recovered in the distantly related subfamily Parreysiinae. *Oxynaia jourdyi* is resolved in a shallow and strongly supported clade comprised of representatives of the genus *Nodularia* Conrad, 1853 (BS/BPP = 100). The *Oxynaia pugio* is recovered in a well-supported clade with the genus *Radiatula* Simpson, 1900. The genus *Indonaia* Prashad, 1918 appears to be the closest relative of the *Oxynaia pugio* + *Radiatula* clade, but with only moderate support values (BS = 61; BPP = 81).

### Morphological analyses

**Figure 3:**
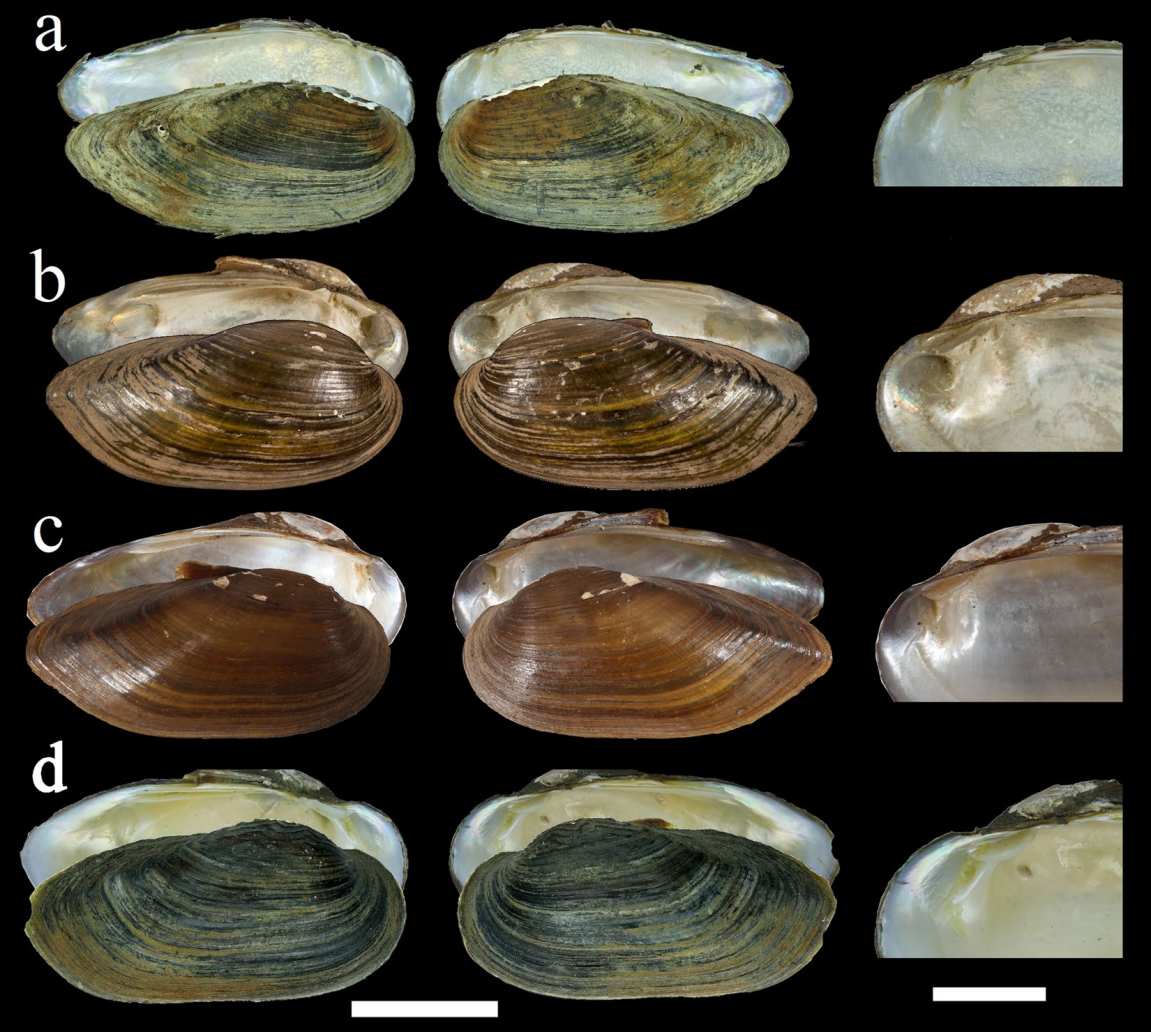
Shell morphology and hinge plate of species in the genera *Indochinella* Bolotov, Pfeiffer, Vikhrev & Konopleva **gen. nov**., *Oxynaia* Haas, 1911, and *Nodularia* Conrad, 1853. (**a**) *Indochinella pugio* (Benson, 1862) **gen. et comb. nov**., Nant Phar Lake, Irrawaddy River basin, Myanmar (RMBH no. biv_258_1). (**b**) *Oxynaia jourdyi* (Morlet, 1886) (syntype MNHN-IM-2000-33685). (**c**) *O. jourdyi*, our sequenced specimen, northern Vietnam (UF 507885). (**d**) *Nodularia douglasiae* (Griffith & Pidgeon, 1833), Soldatskoe Lake, Razdolnaya River basin, Russian Far East (RMBH no. biv_227_12). Scale bars: 2 cm (shells) and 1 cm (hinge plates). (Photos: Ekaterina S. Konopleva (**a**,**d**), Manuel Caballer (**b**) MNHN, Program RECOLNAT, no. ANR-11-INBS-0004], and John M. Pfeiffer (**c**).

Comparisons of the soft anatomy and larval morphology of representatives of the major freshwater mussel clades in the Oriental Region clearly demonstrate the polyphyly of *Oxynaia* s. lato (Table 1). The *Oxynaia jourdyi* group is unambiguously placed in the subfamily Unioninae by the combination of ectobranchous brooding condition and hooked larvae with basal spines, known synapomorphies of the Unioninae^3,5,16^. The *Oxynaia pugio* group (=*Indochinella* **gen. nov**.) is united with all other members of the subfamily Parreysiinae on the basis of possessing a tetragenous brooding condition (sans Lamellidentini Modell, 1942 = ectobranchous), unhooked glochidia, as well as having the ascending lamella of the inner demibranchs attached to the visceral mass across their entire length (vs. attached only anteriorly in the Unioninae). This combination of traits is a novel method of recognizing the Parreysiinae (sans Lamellidentini). Several shell characters also unite the *Oxynaia jourdyi* group with representatives of the Unioninae, especially *Nodularia*, including the position, elevation, and sculpturing of the umbo, as well as several features of the dentition (Table 2 and Fig. 3). *Nodularia* differs from *Indochinella* **gen. nov**. by having a very pronounced and elevated umbo (vs. not pronounced and not elevated), umbo location in the first half of the shell (vs. in the first third), nodulose wrinkles umbo sculpture (vs. v-shaped), and a rectangular and sharp anterior pseudocardinal tooth and a thick and pyramidal posterior pseudocardinal tooth in the left valve (vs. two separated ribbed teeth placed in parallel line with one another).

**Table 1:** List of conchological and anatomical characters in Parreysiinae and other selected subfamilies of the Unionidae.

Taxon	Voucher no.	Inner demibranch ascending lamella fusion to visceral mass	Brooding	Glochidia	Higher classification
*Lamellidens marginalis* (Lamarck, 1819)	CAS180833	Complete	Ectobranchous	Unhooked	Parreysiinae: Lamellidentini
*Parreysia corrugata* (Müller, 1774)	N/A^35,36^	Complete	Tetragenous	Unhooked	Parreysiinae: Parreysiini
*Leoparreysia burmana* (Blanford, 1869)	CAS180831	Complete	Tetragenous	Unhooked	Parreysiinae: Leoparreysiini
*Coelatura* sp.	UF 510905	Complete	Tetragenous	Unhooked	Parreysiinae: Coelaturini
*Indonaia caerulea* (Lea, 1831)	UF507572	Complete	Tetragenous	Unhooked	Parreysiinae: Indochinellini
*Raditula* aff. *humilis* sp.2	UF 507848	Complete	Tetragenous	Unhooked	Parreysiinae: Indochinellini
*Indochinella pugio* (Benson, 1862) **gen. et comb. nov**.	CAS180796, CAS189963	Complete	Tetragenous	Unhooked	Parreysiinae: Indochinellini
*Nodularia douglasiae* (Griffith & Pidgeon, 1833)	RMBH: biv227_12, biv132, biv134	Anterior end	Ectobranchous	Triangular, hooked	Unioninae
*Oxynaia micheloti* (Morlet, 1886)*	NCSM 84920, NCSM 84425	Anterior end	Ectobranchous	Triangular, hooked	Unioninae
*Pyganodon grandis* (Say, 1829)	UF369750	Anterior end	Ectobranchous	Triangular, hooked	Unioninae: Anodontini
*Rectidens sumatrensis* (Dunker, 1852)	UF410001	Anterior end	Ectobranchous	n/a	Rectidentinae: Rectidentini
*Contradens contradens* (Lea, 1838)	UF507874, UF507591	Anterior end	Ectobranchous	Asymmetrical	Rectidentinae: Contradentini
*Monodontina vondembuschiana* (Lea, 1840)	UF507565, UF507438	Anterior end	n/a	n/a	Pseudodontinae: Pilsbryoconchini
*Pilsbryoconcha* sp.	UF507453	Anterior end	Tetragenous	Unhooked	Pseudodontinae: Pilsbryoconchini
*Chamberlainia hainesiana* (Lea, 1856)	UF507722, UF507872	Complete	n/a	n/a	Gonideinae: Chamberlainiini

n/a – not available. *We used this species as a representative of the *Oxynaia jourdyi* group, because gravid individuals of the type species of this genus were not available.

**Table 2:** Comparative analysis of the genera *Indochinella* Bolotov, Pfeiffer, Vikhrev & Konopleva **gen. nov**., *Oxynaia* Haas, 1911, and *Nodularia* Conrad, 1853 on the basis of conchological features.

Conchological features	*Unio pugio* Benson, 1862, the type species of the genus *Indochinella*	*Unio jourdyi* Morlet, 1886, the type species of the genus Oxynaia*	*Unio douglasiae* Griffith & Pidgeon, 1833, the type species of the genus Nodularia**
Shell shape	Cuneiform	Somewhat cuneiform, with more height, anteriorly rounded, posteriorly elongated and pointed	Oval-form, slightly narrow in the posterior part
Umbo	Not pronounced	Very pronounced, elevated	Very pronounced, elevated
Umbo sculpture	V-shaped	Nodulose wrinkles	Nodulose wrinkles
Umbo position	In the first third of the shell	In the first half of the shell	In the first half of the shell
Pseudocardinal teeth of the left valve	Two separated ribbed teeth placed in parallel line with one another	Anterior tooth rectangular and sharp, posterior tooth thick and pyramidal	Anterior rectangular, sharp and ribbed, posterior tooth small and pyramidal
Pseudocardinal teeth of the right valve	Anterior tooth reduced, posterior tooth somewhat pyramidal and wrinkled	Anterior tooth lamella-shaped, posterior tooth rectangular, wrinkled	Anterior tooth reduced, lamella-shaped, posterior tooth somewhat trapeziform and ribbed
Lateral teeth	Rather short, two teeth on the left and one tooth on the right valve	Long, two teeth on the left and one tooth on the right valve	Straight, elongate, sharp with small scratches, two teeth on the left and one tooth on the right valve

*Based on the two syntypes (MNHN-IM-2000-33685, Coll. du Journal de Conchyliologie, ex Coll. Morlet; type locality: Tonkin. Environs de Dang-son (Jourdy) [p. 77]^18^; Bac-Hat, étangs du bord de la rivière Claire (Jourdy) [p. 290]^18^. **Based on a sample from Soldatskoe Lake, Razdolnaya River Basin, Russian Far East (RMBH no. biv_227_12).

The combination of the molecular phylogeny, soft anatomy, larval characters, and shell morphology clearly demonstrate the polyphyly of *Oxynaia* s. lato. The type species and its allies (i.e. the *Oxynaia jourdyi* group) are unambiguously placed in the subfamily Unioninae, rendering both *Oxynaia* and Oxynaiini inapplicable to the Parreysiinae. The tribe Oxynaiini Starobogatov, 1970 is herein recognized as an available family-group level name of the Unioninae Rafinesque, 1820. In the absence of an available name for the *Oxynaia pugio* species group and the larger Parreysiinae clade including the *O. pugio* group, *Radiatula*, and *Indonaia* (i.e. the former Oxynaiini), the genus *Indochinella* **gen. nov** and tribe Indochinellini **trib. nov**. are described here.

### Range disjunction

The two species groups of *Oxynaia* have clearly distinct ranges (Fig. 1). All the reliable records (mostly type localities, see Taxonomic Account) of the *Oxynaia jourdyi* species group are concentrated within the Red, Cả and Cầu River drainage basins of northern Vietnam. The *Oxynaia pugio* species group is known from the Irrawaddy, Sittaung and Tavoy River drainages. Neither species group occurs in the drainages situated between eastern Myanmar and northern Vietnam (i.e., Salween, Mae Klong, Chao Phraya, and Mekong). Zieritz *et al*.^17^ listed two species, *Oxynaia gladiator* and *O. micheloti*, from the Mekong River but those reports refer to misidentified specimens (MNHN IM-2014-6880) or suspect localities (FMNH 20402 and NCSM 84425).

**Taxonomic Account**

**Family Unionidae Rafinesque, 1820**

**Subfamily Unioninae Rafinesque, 1820**

Type genus: *Unio* Philipsson in Retzius, 1788

**Genus** ***Nodularia*** **Conrad, 1853**

Type species: *Unio douglasiae* Griffith & Pidgeon, 1833 (by original designation)

Type locality: Unknown.

= *Oxynaia* Haas, 1911 **syn. nov**. [Type species: *Unio jourdyi* Morlet, 1886]

**Comments:** We recognize *Oxynaia* as a junior synonym of *Nodularia*, and transfer the four Vietnamese species previously treated as *Oxynaia* to *Nodularia* as well. Additionally, we considered *Nodularia dorri* as the fifth member of this group. The close geographic proximity of the type localities and the conchological similarity of the named taxa raises questions about their validity and deserves further research.

*Nodularia jourdyi* (Morlet, 1886) **comb. res**.

*Unio jourdyi* Morlet (1886): p. 76^18^.

*Nodularia jourdyi* Simpson (1900): p. 816^19^.

*Oxynaia jourdyi* Haas (1911): Pl. 16^20^; Haas (1913): p. 152^15^.

**Type locality:** Tonkin. Environs de Dang-son [Đặng Sơn] (Jourdy) [p. 77]^18^; Bac-Hat [Bắc Hà], étangs du bord de la rivière Claire (Jourdy) [p. 290]^18^.

**Distribution:** Red and Cả River drainage basins, northern Vietnam.

*Nodularia gladiator* (Ancey, 1881) **comb. res**.

*Unio gladiator* Ancey (1881): p. 468^21^.

*Oxynaia micheloti* Haas (1913): p. 156^15^.

*Nodularia gladiator* Simpson (1914): p. 991^22^.

**Type locality:** Yon-Bag, Tonkin^21^.

**Distribution:** Red River drainage basin, northern Vietnam. Record from the Mekong Basin^17^ is erroneous.

*Nodularia diespiter* (Mabille, 1887) **comb. res**.

*Unio diespiter* Mabille (1887): p. 162^23^; Simpson (1900): p. 861^19^.

*Nodularia diespiter* Simpson (1914): p. 993^22^.

*Oxynaia diespiter* Haas (1911): Pl. 15^20^; Haas (1913): p. 154^15^.

**Type locality:** Tonkin^23^.

**Distribution:** Red River drainage basin, northern Vietnam.

*Nodularia micheloti* (Morlet, 1886) **comb. res**.

*Unio micheloti* Morlet (1886): p. 77^18^.

*Nodularia micheloti* Simpson (1900): p. 814^19^.

*Oxynaia micheloti* Haas (1911): Pl. 14^20^; Haas (1913): p. 156^15^.

**Type locality:** Tonkin, environs de Chu^18^.

**Distribution:** Cầu River drainage basin, northern Vietnam. Record from the Mekong Basin^17^ is erroneous.

*Nodularia dorri* (Wattebled, 1886)

*Unio dorri* Wattebled (1886): p. 71^24^.

*Nodularia dorri* Simpson (1900): p. 809^19^.

**Type locality:** Les arroyos des environs de Hué^24^.

**Distribution:** Perfume River, central Vietnam. Zieritz *et al*.^17^ erroneously listed *N. dorri* as an endemic species of the Mekong Basin.

**Subfamily Parreysiinae Henderson, 1935**

**Type genus:** *Parreysia* Conrad, 1853

**Comments:** This subfamily includes five tribes: Parreysiini Henderson, 1935, Coelaturini Modell, 1942, Lamellidentini Modell, 1942, Leoparreysiini Vikhrev, Bolotov & Kondakov, 2017^7^ and Indochinellini **trib. nov**., a new tribe described here.

**Tribe Indochinellini Bolotov, Pfeiffer, Vikhrev & Konopleva trib. nov**.

Type genus: *Indochinella* Bolotov, Pfeiffer, Vikhrev & Konopleva **gen. nov**.

Figures 3a and 4

**Figure 4:**
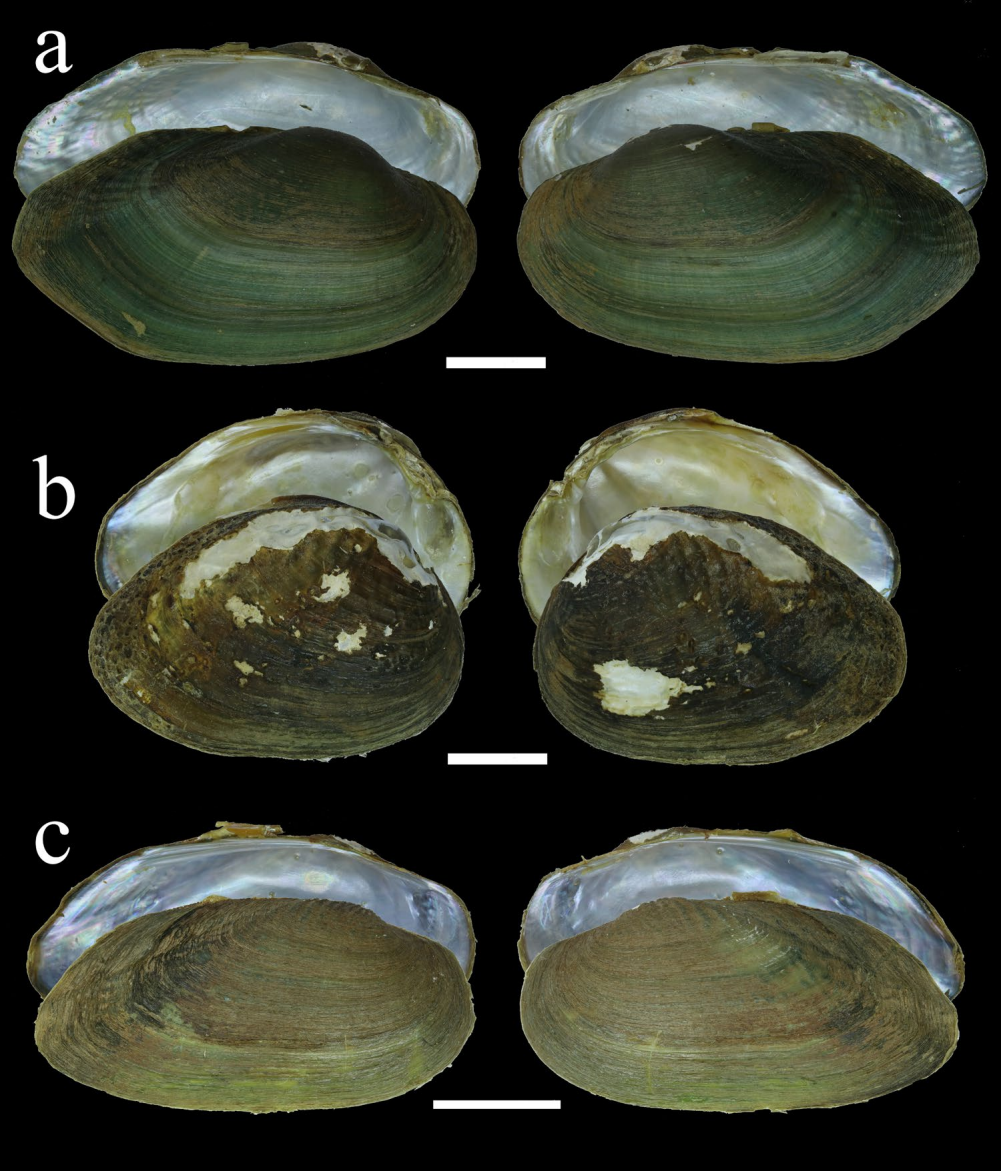
Shell morphology of additional representatives in the tribe Indochinellini. (**a**) *Indonaia andersoniana* (Nevill, 1877), Myaung Lake, Irrawaddy River basin, Myanmar (RMBH no. biv267_1). (**b**) *Radiatula myitkyinae* (Prashad, 1930), Indawgyi Lake, Irrawaddy River basin, Myanmar (RMBH no. biv107_2). (**c**) *Radiatula* cf. *humilis* (Lea, 1856), Chi River near Maha Sarakham, Thailand (RMBH no. biv129_1). Scale bars = 1 cm. (Photos: Ekaterina S. Konopleva).

**Diagnosis:** The Parreysiini and Leoparreysiini are the most morphologically similar family-group level taxa to the Indochinellini. However, the Indochinellini can be distinguished from the Parreysiini and Leoparreysiini by having a much more elongate shell outline (vs. circular) and a nearly straight to convex ventral margin (vs. strongly convex).

**Description:** Adults small (22 mm) to medium (54 mm) sized for family. Shell outline narrow, elongate, strongly inequilateral, always with a straight or convex ventral margin. Moderately inflated, posterior ridge rounded with moderately to very steep posterior slope. Umbo only slightly elevated above hinge line usually with v-shaped umbo sculpture. Green zigzag sculpturing on shell disc common, but absent in some individuals and taxa, e.g., *Indonaia caerulea* (Lea, 1831), *Radiatula humilis* (Lea, 1856), *R. pilata* (Lea, 1866), and *Indochinella pugio* **gen. et comb. nov**. Shells moderately thick. Pseudocardinals erect and stumpy to long and bladelike; two in left valve (may become one in blade-like teeth) and one in right valve occasionally with a second rudimentary anterior tooth. Laterals are moderately short and diverging; two in left, one in right. Unhooked glochidia brooded in all four gills. Mantle margin ventral to incurrent aperture with many prominent simple papillae. Incurrent aperture papilose, excurrent and supra-anal apertures smooth. Ascending lamella of inner demibranch attached to visceral mass for its entire length.

**Distribution:** Southeast and South Asia^6,7^.

**Comments:** This tribe includes at least three valid genera: *Indochinella* **gen. nov**., *Radiatula* Simpson, 1900, and *Indonaia* Prashad, 1918.

**Genus** ***Indochinella*** **Bolotov, Pfeiffer, Vikhrev & Konopleva gen. nov**.

Type species: *Unio pugio* Benson, 1862.

Type locality: Regione Ava [Mandalay]^25^.

Figure 3a, Tables 1 and 2

**Etymology:** The name of this genus is derived from the greater Indochinese Peninsula.

**Diagnosis:** The genus is distinguished from *Nodularia* by the presence of tetragenous brooding of unhooked glochidia (vs. ectobranchous brooding of hooked glochidia), as well as complete fusion of the ascending lamella of the inner demibranchs to the visceral mass (vs. only anterior fusion) (Table 1). *Indochinella* **gen. nov**. can also be distinguished from *Nodularia* by the umbo being only slightly elevated above the hinge line (vs. strongly elevated), fine v-shaped beak sculpture (vs. wrinkled and nodular), triangular posterior pseudocardinal (vs. trapezoidal or rectangular), and moderately short lateral teeth (vs. elongate) (Table 2 and Fig. 3). Adult *Indochinella* (with exception of a lineage from the Tavoy River) can be distinguished from all other representatives of the tribe by the presence of a sharp posterior ridge (vs. rounded) and very steep posterior slope (vs. gradual). Pseudocardinal teeth in Indochinella also tend to be more strongly developed than in other Indochinellini.

**Description:** Shell moderately thick; elongate with rounded anterior end and strongly pointed posterior end. Posterior ridge sharp. Posterior slope steep. Umbo hardly elevated above hinge line, fine v-shaped umbo sculpture, shell disc smooth to strongly sculptured. Moderately inflated. Pseudocardinal teeth strong, two in the left valve, one in the right. Laterals moderately short, two in left valve, one in right. Tetragenous brooding condition (occasionally ectobranchous), unhooked glochidia.

**Distribution:** The genus is primarily known from Myanmar in the Irrawaddy, Sittaung, and Tavoy River drainages. The genus may also inhabit several other river basins in Myanmar, e.g., the Great Tenasserim, Salween, and some coastal rivers of the Bay of Bengal^14,26–28^. A few records from India (e.g., Assam)^14^ are in need of future studies because these specimens could have been collected within the Irrawaddy Basin.

**Comments:** We assigned a single described species to the genus, although the divergent molecular lineages from the Sittaung and Tavoy River drainage basins may be worthy of formal taxonomic recognition.

*Indochinella pugio* (Benson, 1862) **gen. et comb. nov**.

*Unio pugio* Benson (1862): p. 193^25^.

*Nodularia pugio* Simpson (1900): p. 814^19^.

*Oxynaia pugio* Haas (1911): Pl. 14^20^; Haas (1913): p. 158^15^.

Figures 1 and 3a, Tables 1 and 2

**Material examined:** Myanmar: Irrawaddy River basin, Nant Phar Lake, 24.2972°N, 97.2610°E, 29.xi.2016, 4 specimens (RMBH nos. biv_258_1, biv_258_2, biv_258_4, and biv_258_5), Vikhrev leg. Myanmar: Irrawaddy River basin, Myaung Lake, 24.2387°N, 97.1658°E, 01.xii.2016, 4 specimens (RMBH nos. biv_268, biv_268_1, biv_268_2, and biv_268_4), Vikhrev leg. Myanmar: Irrawaddy River basin, Pauk In Lake, 21.81347°N, 95.19746°E, 13.x.2009, 5 specimens (CAS 180788), Pitotrowski *et al*. leg. Myanmar: Irrawaddy River basin, Irrawaddy River near Myingyan, 21.48187°N, 95.30501°E, 15.x.2009, 5 specimens (CAS 189963), Pitotrowski *et al*. leg., Myanmar: Irrawaddy River basin, Chindwin River near confluence with Irrawaddy, 21.498445°N, 95.26631°E, 09.x.2009, 5 specimens (CAS 180796), Pitotrowski *et al*. leg. Myanmar: Sittaung River basin, Myit Kyi Pauk Stream, 18.9613°N, 96.4455°E, 26.xi.2016, 3 specimens (RMBH nos. biv_251_3, biv_251_1, and biv_251_2), Vikhrev leg. Myanmar: Tavoy River, 14.5012°N, 98.1557°E, 26.iv.2015, 26.iv.2015, 38 specimens (RMBH nos. biv_147_10, biv_147_3, biv_147_18, biv_148_4, biv_148_7, and biv_148_15 are sequenced), Bolotov leg.

**Redescription:** Shell shape cuneiform, elongated, inequilateral, not inflated, rather thick. Posterior ridge sharp, posterior slope steep. Maximum shell length 53.4 mm, height to 24.3 mm, width to 18.7 mm. Fine v-shaped sculpture on umbo, umbo only slightly elevated above hinge line. Periostracum smooth, grey-brown to yellow-green, with dark parts along the radial lines; nacre whitish. Left valve with two parallel rather short lateral teeth and two ribbed parallel pseudocardinal teeth. Right valve with a single slightly curved lateral tooth and two pseudocardinal teeth, anterior tooth reduced, posterior tooth high, ribbed and strong. Umbo cavity not very deep, nacre in umbo cavity commonly tinted peach to golden-brown. Anterior adductor scar well pronounced, funneled; posterior adductor scar rounded. The Sittaung lineage differs from the Irrawaddy lineage in having a shorter and higher shell, more pronounced and curved lateral teeth, and a moderately strong sculpture on shell disc. The Tavoy lineage differs from the two other lineages in having an oval-shaped shell, more rounded posterior ridge, more gradual posterior slope, and distinct zigzag ridges across shell disc.

**Distribution:** Irrawaddy, Sittaung and Tavoy River basins. In the Irrawaddy River, it is known as far north as Mya Taung and as far south as Hinthada. A few records from India (e.g., Assam)^14^ are in need of future studies because these specimens could have been collected within the Irrawaddy Basin. The lineages from the Sittaung and Tavoy River catchment areas may represent separate species- or subspecies-level taxa but requires further systematic research.

**Habitat and ecology:** The species seems to be rather a habitat generalist, and it is known from the mainstream of large, medium-sized and small rivers, as well as from their floodplain lakes.

**Comments:** *Unio digitiformis* Sowerby, 1868 from India is not a synonym of *Indochinella pugio* **gen. et comb. nov**^29^. but a separate species, the generic placement of which is unclear. Haas^13^ noted that the location of this form is certainly not India and that it most likely belong to the *Lanceolaria* Conrad, 1853.

## Discussion

### Taxonomic implications

Our integrative molecular and morphological approach has determined that the genus *Oxynaia* is polyphyletic. This result has clear implications regarding the higher-level classification of the Unionidae, the morphological characteristics of the Parreysiinae, and corroborates broader biogeographic patterns in Southeast Asia. Molecular and morphological data reject the monophyly of *Oxynaia* with its former constituents being recovered in phylogenetically divergent and morphologically diagnosable clades. The *Oxynaia jourdyi* group is unambiguously placed in the Unioninae on the basis of our molecular phylogeny and several morphological synapomorphies and is herein considered a junior synonym of *Nodularia*, rendering the tribe name Oxynaiini inapplicable to the Parreysiinae. This taxonomic rearrangement required the description of a new genus for the “*Oxynaia*” *pugio* group (=*Indochinella* **gen. nov**.) and a new tribe (=Indochinellini **trib. nov**.) to recognize these morphologically cohesive clades of Parreysiinae.

### Freshwater biogeography of Southeast Asia

**Figure 5:**
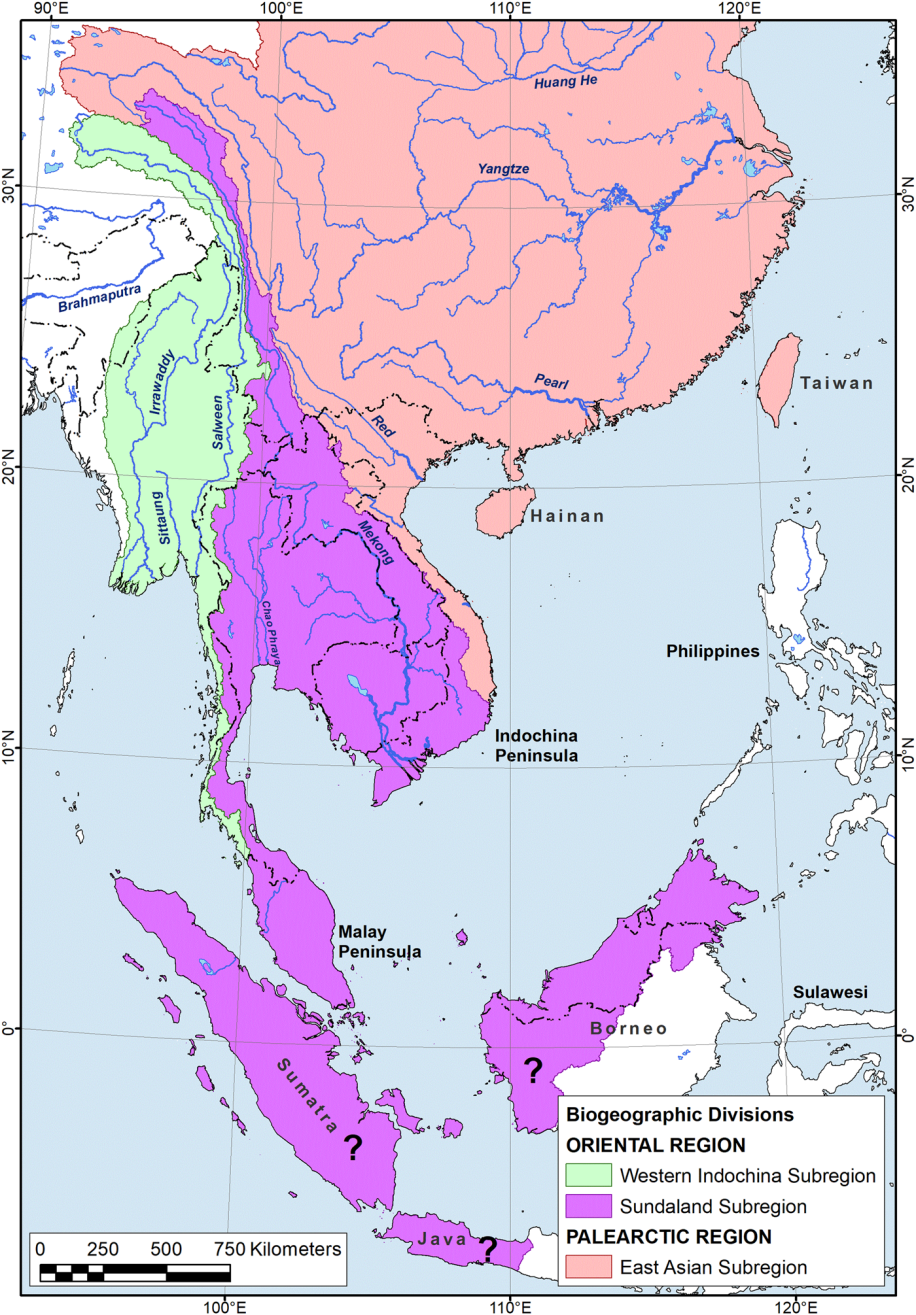
Freshwater biogeographic division of Southeast Asia based on the phylogeny and phylogeography of the Unionidae^5–9^. The question marks indicate areas that were tentatively assigned by us to the Sundaland Region but their placement is in need of future research. The map was created using ESRI ArcGIS 10 software (www.esri.com/arcgis); the topographic base of the map was created with Natural Earth Free Vector and Raster Map Data (www.naturalearthdata.com). (Map: Mikhail Yu. Gofarov).

Previous to this study, *Oxynaia* was thought to be one of the few genera distributed across much of Southeast Asia from central Myanmar to northeastern Vietnam^17^. This distribution was thought to be unusual in that it crossed two important biogeographic barriers in Southeast Asia: (1) the Salween/Mekong river drainage divide separating the Western Indochinese freshwater mussel assemblage from the Sundaland assemblage, and (2) the Mekong/northern Vietnamese drainage divides separating the Sundaland assemblage from the East Asian fauna. However, the seemingly large distribution of *Oxynaia* s. lato is discovered here to be spurious and based on incorrect interpretations of common ancestry. The geographic distributions of *Nodularia* and *Indochinella* closely follow these two influential biogeographic barriers. These two biogeographic barriers divide eastern Asia into three faunistically distinct subregions (Fig. 5), each of which is briefly discussed below.

1. Western Indochina Subregion. The drainages of the Arakan coast of Myanmar, the Irrawaddy, Pegu, Sittaung, and Bilin river basins, and east to the Salween River and western drainages of the Kra Isthmus . The subregion currently includes members of three subfamilies (Parreysiinae, Rectidentinae and Pseudodontinae), five tribes and eight genera^6,7^. *Indochinella* **gen. nov**. is the fifth endemic unionid genus of western Indochina, together with *Pseudodon* Gould, 1844, *Trapezoideus* Simpson 1900, *Leoparreysia* Vikhrev, Bolotov & Aksenova, 2017, and *Trapezidens* Bolotov, Vikhrev & Konopleva, 2017. The other genera in the region, *Lamellidens* Simpson, 1900, *Indonaia*, and *Radiatula* are geographically more widespread, especially to the west into India. *Radiatula* is the only genus recognized to occur to the west and east of the Salween River drainage basin^6,7^.
2. Sundaland Subregion. The Mekong, Chao Phraya, Mae Klong and the drainages of the Malay Peninsula, probably corresponding to the gigantic paleo-Mekong River basin^6–8^. The fauna of the Greater Sunda Islands (Sumatra, West Java, northern and western Borneo) appears similar to that of mainland Southeast Asia and may also belong to this subregion, as suggested by the molecular data for northern Borneo^9^ and by the putative connections of paleo-drainages during the Pleistocene^30^, but further systematic research is necessary to delineate these boundaries. The Sundaland Subregion is comprised at least five subfamilies, i.e. Parreysiinae, Rectidentinae, Pseudodontinae, Gonideinae, and the monotypic Modellnaiinae. A single member of the Unioninae (i.e., *Cristaria plicata*)^31,32^ occurs in the region, although, it has likely been introduced. Two large endemic monophyletic radiations of freshwater mussels, i.e., the tribes Pilsbryoconchini and Rectidentini, were recorded in this region^6,7^. The highest levels of diversity of these clades occur within the Mekong River basin, with a few representatives inhabiting the Chao Phraya River, the Malay Peninsula, and the Greater Sunda Islands^6–9^. The Indochinellini is the only tribe of the Parreysiinae distributed in the Sundaland Subregion, whose constituents belong to a subclade *Radiatula* that appears to have significant levels of cryptic diversity (Fig. 2). Several characteristic elements of the fauna of western Indochina are lacking in Sundanese assemblage, such as the members of Lamellidentini, Leoparreysiini, and Pseudodontini.
3. East Asian Subregion. The Red and Ca River drainage basins, and numerous coastal rivers of Vietnam comprise the East Asian Subregion, whose fauna appears to be more closely allied to the Palearctic Region than to the Oriental Region (Fig. 5). This large biogeographic subregion extends north to Japan and the Far East of Russia. This freshwater mussel fauna is entirely different from those of Western Indochina and Sundaland, and it has strong biogeographic affinities to the Pearl, Yangtze and Huang He river drainage basins^33^. With respect to available paleontological data^34^ and phylogenetic modelling^6,7^, the freshwater bivalve faunas of the Mekong and Yangtze have been developing independently since at least the early Cenozoic epoch. The East Asian Unioninae taxa such as the *Nodularia*, *Cristaria* Schumacher, 1817, *Sinanodonta* Modell, 1945, *Lamprotula* Simpson, 1900 and *Sinohyriopsis* Starobogatov, 1970 are the most characteristic elements of the freshwater mussel fauna of eastern Indochina at least since the Eocene^34^, while the Rectidentinae, Parreysiinae and Pseudodontinae appear completely absent there. Based on the patterns outlined above, we suggest that the East Asian Subregion belongs to the Palearctic Region.

This biogeographic division of Southeast Asia largely corresponds with that of Graf and Cummings^33^ suggesting four freshwater biogeographic subregions, i.e. (1) Yangtze-Huang, from the Pei south to the Qiantang and Taiwan; (2) Indochina, including southern China and the Mekong west to the Salween; (3) India–Burma, from the Indus to the Irrawaddy; and (4) Sunda Islands–Philippines. However, our new scheme (Fig. 5) reveals that the Salween, Irrawaddy and Sittaung unionid faunas are close to each other and should belong to the separate Western Indochina Subregion and that the Yangtze-Huang (=East Asian) Subregion comprises the drainage basins in northern Vietnam and appears to be a part of the Palearctic Region. Additionally, we suggest that Sumatra, West Java, northern and western Borneo may belong to the Sundaland Subregion, but this preliminary hypothesis is in need of future confirmation based on an expanded molecular dataset.

Zieritz *et al*.^17^ distinguished two major hotspots (“epicentres”) of the subfamily-level diversity and endemism of the Unionidae in Asia, i.e. (1) Southeast Asian Hotspot harboring the highest diversity of the Rectidentinae, Gonideinae (+Pseudodontinae), Parreysiinae, and Modellnaiinae, and (2) Chinese Hotspot dominated by the Unioninae (+Anodontini) lineages. Our biogeographic division is largely congruent with this diversity-based model, i.e., the Western Indochinese and Sundaland subregions correspond to the Southeast Asian diversity hotspot and the East Asian Subregion correlates with the Chinese diversity hotspot.

## Methods

### Nomenclatural acts

The electronic edition of this article conforms to the requirements of the amended International Code of Zoological Nomenclature (ICZN), and hence the new names contained herein are available under that Code from the electronic edition of this article. This published work and the nomenclatural acts it contains have been registered in ZooBank (http://zoobank.org), the online registration system for the ICZN. The LSID for this publication is: urn:lsid:zoobank.org:pub:C585DACD-6AB1-4692-B68A-50ABA47940A4. The electronic edition of this paper was published in a journal with an ISSN, and has been archived and is available from PubMed Central.

### Studied museum collections

The shell lots were studied in the malacological collections of the National Museum of Natural History, Smithsonian Institution, Washington, DC, USA (NMNH), British Museum of Natural History, London, UK (NHMUK), Muséum National d’Histoire Naturelle, Paris, France (MNHN), Museo Civico di Storia Naturale di Genova, Genoa, Italy (MSNG), California Academy of Natural Sciences (CAS), North Carolina State Museum (NCSM), and Florida Museum of Natural History (UF). Additionally, we accessed the images of the types of several nominal taxa at the MUSSELp Database^29^.

### Morphological methods

Soft anatomy and larval characters were scored for representatives of both *Oxynaia* species groups (Table 1). These three characters were chosen as they have been previously demonstrated to be useful in diagnosing suprageneric clades of freshwater mussels^16^. Character states were observed using a Leica M27s dissecting scope and a Leica DM LB2 compound microscope. Representative taxa relevant to previous classifications of *Oxynaia* were also included, as were other major lineages present in the region (Table 1). *Parreysia corrugata* was scored using literature sources^35,36^, as no soft anatomy for this species was available. A more detailed comparison of the shell characters that distinguish the three most commonly confused genera are provided in Table 2. Umbo character states follow Zieritz *et al*.^37^.

### Phylogenetic analyses

We included novel *Oxynaia jourdyi* sequences in the recent phylogenetic data set of Bolotov *et al*.^7^ to test the monophyly of *Oxynaia*. This data set was simplified to include only one haplotype of each species, with exception of the *Oxynaia pugio* sequences (Supplementary Table 1). Additionally, we excluded several taxa that were represented only by sequences of the COI gene, but *Parreysia* spp. and *Indonaia* spp. from India were left as the members of the Parreysiinae. The sequence alignment of *COI*, 16*S rRNA* and 28*S rRNA* gene fragments was performed separately using the Muscle algorithm implemented in MEGA6^38^. The alignment data sets were joined in a multi-locus alignment. Lacking sites were treated as missing data. We performed maximum likelihood and Bayesian inference phylogenetic analyses using RAxML v. 8.2.6 HPC Black Box^39^ and MrBayes v. 3.2.6^40^, respectively. The settings of the analyses were as described in Bolotov *et al*.^7^. The phylogenetic models were calculated at the San Diego Supercomputer Center through the CIPRES Science Gateway^41^.

### Data availability

The sequences used in this study are available from GenBank. Accession numbers for each specimen are presented in Supplementary Table 1.

## Electronic supplementary material

Supplementary Info

## References

[1] Lopes-Lima M (2018). Conservation of freshwater bivalves at the global scale: diversity, threats and research needs. Hydrobiologia.

[2] Graf DL (2013). Patterns of freshwater bivalve global diversity and the state of phylogenetic studies on the Unionoida, Sphaeriidae, and Cyrenidae. American Malacological Bulletin.

[3] Pfeiffer JM, Graf DL (2015). Evolution of bilaterally asymmetrical larvae in freshwater mussels (Bivalvia: Unionoida: Unionidae). Zoological Journal of the Linnean Society.

[4] Konopleva ES, Bolotov IN, Vikhrev IV, Gofarov MY, Kondakov AV (2016). An integrative approach underscores the taxonomic status of *Lamellidens exolescens*, a freshwater mussel from the Oriental tropics (Bivalvia: Unionidae). Systematics and Biodiversity.

[5] Lopes-Lima M (2017). Phylogeny of the most species-rich freshwater bivalve family (Bivalvia: Unionida: Unionidae): Defining modern subfamilies and tribes. Molecular Phylogenetics and Evolution.

[6] Bolotov IN (2017). Ancient river inference explains exceptional Oriental freshwater mussel radiations. Scientific Reports.

[7] Bolotov IN (2017). New taxa of freshwater mussels (Unionidae) from a species-rich but overlooked evolutionary hotspot in Southeast Asia. Scientific Reports.

[8] Zieritz A (2016). Factors driving changes in freshwater mussel (Bivalvia, Unionida) diversity and distribution in Peninsular Malaysia. Science of the Total Environment.

[9] Zieritz A (2018). Changes and drivers of freshwater mussel diversity and distribution in northern Borneo. Biological Conservation.

[10] Whelan NV, Geneva AJ, Graf DL (2011). Molecular phylogenetic analysis of tropical freshwater mussels (Mollusca: Bivalvia: Unionoida) resolves the position of *Coelatura* and supports a monophyletic Unionidae. Molecular Phylogenetics and Evolution.

[11] Thiele, J. *Handbuch der systematischen Weichtierkunde* (Vol. 2, Part 3), 779–1022 (Jena, Gustav Fischer, 1934).

[12] Modell H (1964). The natural system of the naiads. 3. Archiv für Molluskenkunde.

[13] Haas F (1969). Superfamilia Unionacea. Das Tierreich.

[14] Subba Rao, N.V. *Handbook of freshwater molluscs of India* (Calcutta, 1989).

[15] Haas F (1913). Die Unioniden. Systematisches Conchylien-Cabinet von Martini und Chemnitz.

[16] Graf DL, Cummings KS (2006). Palaeoheterodont diversity (Mollusca: Trigonioida+ Unionoida): what we know and what we wish we knew about freshwater mussel evolution. Zoological Journal of the Linnean Society.

[17] Zieritz A (2018). Diversity, biogeography and conservation of freshwater mussels (Bivalvia: Unionida) in East and Southeast Asia. Hydrobiologia.

[18] Morlet L (1886). Diagnoses Molluscorum novorum Tonkini. Journal de Conchyliologie.

[19] Simpson, C. T. Synopsis of the naiades, or pearly fresh-water mussels. *Proceedings of the United States National Museum* **22**, 501–1044 (1900).

[20] Haas F (2011). Die Unioniden. Systematisches Conchylien-Cabinet von Martini und Chemnitz.

[21] Ancey CF (1881). Coquilles Nouvelles ou peu Connues. Le Naturaliste.

[22] Simpson, C. T. A descriptive catalogue of the naiades, or pearly fresh-water mussels (Parts I-III) (Detroit, 1914).

[23] Mabille J (1887). Sur quelques mollusques du Tonkin. Bulletins de la Société Malacologique de France.

[24] Wattebled G (1886). Description de Mollusques inédits de l’ Annam. Récolte du capitaine Dorr aux environs de Hué. Journal de Conchyliologie.

[25] Benson WH (1862). Descriptions of Indian and Burmese species of the genus *Unio*, Retz. Annals and Magazine of Natural History (Third Series).

[26] Tapparone-Canefri C (1889). Viaggio de Leonardo Fea in Birmania e regioni vicine. XVIII. Molluschi terrestri e d’acqua dolce. Annali del Museo Civico di Storia Naturale de Genova (series 2).

[27] Preston, H. B. *Mollusca (Freshwater Gastropoda & Pelecypoda)*. *Fauna of British India, including Ceylon and Burma* (London, Taylor & Francis, 1915).

[28] Prashad B (1922). A revision of the Burmese Unionidae. Records of the Indian Museum.

[29] Graf, D. L. & Cummings, K. S. The freshwater mussels (Unionoida) of the World (and other less consequential bivalves), updated 5 December 2017. MUSSEL Project Web Site. Available: http://www.mussel-project.net (2017).

[30] Voris HK (2000). Maps of Pleistocene sea levels in Southeast Asia: shorelines, river systems and time durations. Journal of Biogeography.

[31] Brandt RAM (1974). The non-marine aquatic mollusca of Thailand. Archiv für Mollusckenkunde.

[32] Nahok B, Tumpeesuwan C, Srifa A, Tumpeesuwan S (2017). Freshwater molluscan assemblages in upper part of Choen River Basin, Northeastern Thailand. Tropical Natural History.

[33] Graf DL, Cummings KS (2007). Review of the systematics and global diversity of freshwater mussel species (Bivalvia: Unionoida). Journal of Molluscan Studies.

[34] Schneider S, Böhme M, Prieto J (2013). Unionidae (Bivalvia; Palaeoheterodonta) from the Palaeogene of northern Vietnam: exploring the origins of the modern East Asian freshwater bivalve fauna. Journal of Systematic Palaeontology.

[35] Ortmann AE (1910). The systematic position of the unionid genus *Parreysia*. Nautilus.

[36] Prashad B (1918). Studies on the anatomy of Indian Mollusca, II. The marsupium and glochidium of some Unionidae and on the Indian species hitherto assigned to the genus *Nodularia*. *Records of the Indian*. Museum.

[37] Zieritz A, Sartori AF, Bogan AE, Aldridge DC (2015). (2015). Reconstructing the evolution of umbonal sculptures in the Unionida. Journal of Zoological Systematics and Evolutionary Research.

[38] Tamura K, Stecher G, Peterson D, Filipski A, Kumar S (2013). MEGA6: Molecular Evolutionary Genetics Analysis version 6.0. Molecular Biology and Evolution.

[39] Stamatakis A (2006). RAxML-VI-HPC: maximum likelihood-based phylogenetic analyses with thousands of taxa and mixed models. Bioinformatics.

[40] Ronquist F (2012). MrBayes 3.2: Efficient Bayesian Phylogenetic Inference and Model Choice Across a Large Model Space. Systematic Biology.

[41] Miller, M., Pfeiffer, W. & Schwartz, T. Creating the CIPRES Science Gateway for inference of large phylogenetic trees. In Gateway Computing Environments Workshop (GCE). 1–8 (IEEE, 2010).

