# Supplementary material for: A new genus and tribe of freshwater mussel (Unionidae) from Southeast Asia
Source: Sci Rep. 2018 Jul 3;8:10030. doi: 10.1038/s41598-018-28385-y (PMC6030202; doi:10.1038/s41598-018-28385-y)
Supplement: Supplementary file 1 — Supplementary Info [file 41598_2018_28385_MOESM1_ESM.doc]

SUPPLEMENTARY INFORMATION

A new genus and tribe of freshwater mussel (Unionidae) from Southeast Asia

Ivan N. Bolotov*, John M. Pfeiffer, Ekaterina S. Konopleva, Ilya V. Vikhrev, Alexander V. Kondakov, Olga V. Aksenova, Mikhail Yu. Gofarov, Sakboworn Tumpeesuwan & Than Win

*Corresponding author: inepras@yandex.ru

**Supplementary Table 1.** List of sequences used in this study, including the species, the location and NCBI’s GenBank accession numbers

| **Taxa** | **Locality** | **Code** | **COI** | **28S rDNA** | **16S** |
| --- | --- | --- | --- | --- | --- |
| In-Group Taxa |  |  |  |  |  |
| **UNIONIDAE Rafinesque, 1820** |  |  |  |  |  |
| **PARREYSIINAE Henderson 1935** |  |  |  |  |  |
| **Lamellidentini Modell, 1942** |  |  |  |  |  |
| *Trapezidens exolescens* (Gould, 1843) | Myanmar: Tavoy River | TraExo | KX230532 | KX230559 | KX230548 |
| *T. dolichorhynchus* (Tapparone Canefri, 1889) | Myanmar: Irrawaddy River | TraDol | JN243903 | JN243881 | KP795042 |
| *Lamellidens savadiensis* (Nevill, 1877) | Myanmar: Irrawaddy River basin, Lake Indawgyi | LamSav | KX230544 | KX230566 | KX230555 |
| *L. brandti* Bolotov, Konopleva & Vikhrev, 2017 | Myanmar: Sittaung River basin, Pathi river | LamBra | MF352219 | MF352351 | MF352293 |
| *L. generosus* (Gould, 1847) | Myanmar: Salween River basin, Lake Inle | LamGen | KX865825 | KX865699 | KX865596 |
| *L.* aff. *marginalis* (Lamarck, 1819) sp.3 | Myanmar: Kaladan River basin, unnamed puddle | LamMar | KX230547 | KX230569 | KX230558 |
| **Parreysiini Henderson, 1935** |  |  |  |  |  |
| *Parreysia* cf. *corrugata* (Müller, 1774) sp.1 | India: Periyar River | ParCor1 | KJ872809 | n/a | n/a |
| *P.* cf. *corrugata* (Müller, 1774) sp.2 | India: Krishna River basin, Ghataprabha River | ParCor2 | JQ861229 | n/a | n/a |
| **Leoparreysiini Vikhrev, Bolotov & Kondakov, 2017** |  |  |  |  |  |
| *Leoparreysia olivacea* (Prashad, 1930) | Myanmar: Irrawaddy River | LeoOli | KP795022 | KP795005 | KP795044 |
| *L. canefrii* Vikhrev, Bolotov & Kondakov, 2017 | Myanmar: Sittaung River near Taungoo | LeoCan | MF352237 | MF352365 | MF352307 |
| *L. tavoyensis* (Gould, 1843) | Myanmar: Tavoy River | LeoTav | KX230543 | KX230565 | KX230554 |
| **Coelaturini Modell, 1942** |  |  |  |  |  |
| *Nitia teretiuscula* (Philippi, 1847) | Egypt: Nile River | NitTer | JN243897 | JN243875 | n/a |
| *Coelatura* aff. *aegyptiaca* (Cailliaud, 1827) sp.1 | Egypt: Nile River | CoeAeg | JN243894 | JN243872 | KP795045 |
| **Indochinellini trib. nov.** |  |  |  |  |  |
| *Indochinella pugio* (Benson, 1862) **gen. et comb. nov.** | Myanmar: Irrawaddy River basin, Lake Nant Phar | IndPug | MF352261 | MF352386 | MF352325 |
| *I. pugio* (Benson, 1862) **gen. et comb. nov.** | Myanmar: Irrawaddy River basin, Lake Nant Phar | IndPug | MF352262 | MF352389 | MF352326 |
| *I. pugio* (Benson, 1862) **gen. et comb. nov.** | Myanmar: Irrawaddy River basin, Lake Myaung | IndPug | MF352285 | n/a | n/a |
| *I. pugio* (Benson, 1862) **gen. et comb. nov.** | Myanmar: Irrawaddy River basin, Lake Myaung | IndPug | MF352286 | MF352403 | MF352346 |
| *I. pugio* (Benson, 1862) **gen. et comb. nov.** | Myanmar: Irrawaddy River basin, Lake Myaung | IndPug | MF352287 | MF352404 | MF352347 |
| *I. pugio* (Benson, 1862) **gen. et comb. nov.** | Myanmar: Irrawaddy River basin, Chindwin River: Paukin Lake | IndPug | JN243899 | JN243879 | KP795046 |
| *I. pugio* (Benson, 1862) **gen. et comb. nov.** | Myanmar: Tavoy River | IndPugTV | KX865852 | KX865724 | KX865623 |
| *I. pugio* (Benson, 1862) **gen. et comb. nov.** | Myanmar: Tavoy River | IndPugTV | KX865853 | KX865725 | KX865624 |
| *I. pugio* (Benson, 1862) **gen. et comb. nov.** | Myanmar: Tavoy River | IndPugTV | KX865854 | KX865726 | KX865625 |
| *I. pugio* (Benson, 1862) **gen. et comb. nov.** | Myanmar: Tavoy River | IndPugTV | KX865855 | KX865727 | KX865626 |
| *I. pugio* (Benson, 1862) **gen. et comb. nov.** | Myanmar: Tavoy River | IndPugTV | KX865856 | KX865728 | KX865627 |
| *I. pugio* (Benson, 1862) **gen. et comb. nov.** | Myanmar: Tavoy River | IndPugTV | KX865857 | KX865729 | KX865628 |
| *I. pugio* (Benson, 1862) **gen. et comb. nov.** | Myanmar: Sittaung River basin, Myit Kyi Pauk stream | IndPugST | MF352242 | MF352370 | MF352312 |
| *I. pugio* (Benson, 1862) **gen. et comb. nov.** | Myanmar: Sittaung River basin, Myit Kyi Pauk stream | IndPugST | MF352243 | MF352371 | MF352313 |
| *I. pugio* (Benson, 1862) **gen. et comb. nov.** | Myanmar: Sittaung River basin, Myit Kyi Pauk stream | IndPugST | MF352244 | MF352372 | MF352314 |
| *Indonaia caerulea* (Lea, 1831) | India: Krishna River basin, fish pond | IndCoe | KT869141 | n/a | n/a |
| *I. andersoniana* (Nevill, 1877) | Myanmar: Irrawaddy River basin, Lake Indawgyi | IndAnd | KX865835 | KX865709 | KX865606 |
| *I.* aff. *khadakvaslaensis* (Ray, 1966) sp.1 | India | IndKha | KF690124 | n/a | n/a |
| *I. lima* (Simpson, 1900) | India: Krishna River, Nagarjuna Sagar | IndLim | KP268827 | n/a | n/a |
| *Radiatula mouhoti* Vikhrev, Bolotov & Konopleva, 2017 | Myanmar: Sittaung River near Taungoo | RadMou | MF352234 | MF352363 | MF352305 |
| *R.* cf. *bonneaudii* (Eydoux, 1838) sp.1 | Myanmar: Irrawaddy River basin, Tar Pein River | RadBon | MF352266 | MF352390 | MF352330 |
| *R. myitkyinae* (Prashad, 1930) | Myanmar: Irrawaddy River basin, Lake Indawgyi | RadMyi | KX865838 | KX865710 | KX865609 |
| *R.* *humilis* (Lea, 1856) | Thailand: Mekong River basin, Chi River | RadHum1 | KX865844 | KX865716 | KX865615 |
| *R.* aff. *humilis* (Lea, 1856) sp.2 | Thailand: Mekong River basin, Chi River | RadHum2 | KX865850 | KX865722 | KX865621 |
| *R.* aff. *humilis* (Lea, 1856) sp.3 | Cambodia: Mekong River basin | RadHum3 | KP795023 | KP795006 | KP795048 |
| **PSEUDODONTINAE Frierson, 1927** |  |  |  |  |  |
| **Pseudodontini Frierson, 1927** |  |  |  |  |  |
| *Pseudodon avae* (Theobald, 1873) | Myanmar: Irrawaddy River basin, a tributary of Lake Indawgyi | PseAva | KX865858 | KX865730 | KX865629 |
| *P. bogani* Bolotov, Kondakov & Konopleva, 2017 | Myanmar: Sittaung River basin, Kanni River | PseBog | MF352216 | MF352348 | MF352290 |
| *P. manueli* Konopleva, Kondakov & Vikhrev, 2017 | Myanmar: Sittaung River basin, Pyowne River | PseMan | MF352228 | MF352358 | MF352300 |
| **Pilsbryoconchini Bolotov, Vikhrev & Tumpeesuwan, 2017** |  |  |  |  |  |
| *Bineurus* aff. *mouhotii* (Lea, 1863) sp.1 | Laos: Mekong River basin, Nam Long River | BinMou1 | KX865876 | KX865747 | KX865647 |
| *B.* aff. *mouhotii* (Lea, 1863) sp.2 | Thailand: Mekong River basin, Loei River | BinMou2 | KX865879 | KX865750 | KX865650 |
| *B.* aff. *mouhotii* (Lea, 1863) sp.3 | Laos: Mekong River basin | BinMou3 | KP795026 | KP795009 | KP795051 |
| *Monodontina cambodjensis* (Petit de la Saussaye, 1865) | Cambodia: Mekong River basin, Tonle Sap River: Pursat River | MonCam | KP795028 | KP795011 | KF011262 |
| *M.* aff. *vondembuschiana* (Lea, 1840) sp.1 | Thailand: Mekong River basin, Phong River | MonVon1 | KX865861 | KX865733 | KX865632 |
| *M.* aff. *vondembuschiana* (Lea, 1840) sp.2 | Laos: Mekong River basin | MonVon2 | KP795029 | AF400694 | KP795052 |
| *Pilsbryoconcha compressa* (Martens, 1860) | Thailand: Mekong River basin, artificial pond near the Ban Nong-Bua village | PilCom | KX865872 | KX865744 | KX865643 |
| *P.* aff. *exilis* (Lea, 1838) sp.1 | Cambodia: Mekong River basin | PilExi1 | KP795024 | KP795007 | KP795049 |
| **RECTIDENTINAE Modell, 1942** |  |  |  |  |  |
| **Contradentini Modell, 1942** |  |  |  |  |  |
| *Contradens contradens* (Lea, 1838) | West Malaysia: Pahang River | ConCon | DQ191411 | AF400692 | n/a |
| *C.* aff. *contradens* (Lea, 1838) sp.1 | Cambodia: Mekong River basin | ConCon1 | KP795034 | KP795016 | KP795054 |
| *C. eximius* (Lea, 1856) | Thailand: Mekong River basin, Chi River | ConExi | KX865936 | KX865807 | n/a |
| *C.* sp.'Nam Long' | Thailand: Mekong River basin, Loei River | ConSpNL | KX865928 | KX865799 | KX865682 |
| *C.* sp.'Vieng Phou Kha' | Laos: Mekong River basin, a tributary of Nam Fa River near Vieng Phou Kha | ConSpVPK | KY561630 | KY561662 | KY561645 |
| *Physunio modelli* Brandt, 1974 | Thailand: Mekong River basin, Chi River | PhyMod | KX865883 | KX865754 | KX865654 |
| *P.* sp.'Contradens' | Cambodia: Mekong River basin | PhySpCON | KP795035 | KP795017 | KP795055 |
| *P.* sp.'Trapezoideus' | Laos: Mekong River basin, Nam Ou River | PhySpTRA | KP795036 | KP795018 | KF011265 |
| *Trapezoideus nesemanni* Konopleva, Vikhrev & Bolotov, 2017 | Myanmar: Sittaung River basin, Tauk Ue Kupt River | TraNes | KX865906 | KX865777 | KX865663 |
| *T. panhai* Konopleva, Bolotov & Kondakov, 2017 | Myanmar: Sittaung River basin, Kyan Hone River | TraPan | KX865909 | KX865780 | KX865666 |
| *T.* sp.'Salween' | Myanmar: Salween River basin, Lake Inle | TraSpSW | KX865915 | KX865786 | KX865672 |
| **Rectidentini Modell, 1942** |  |  |  |  |  |
| *Ensidens* aff. *sagittarius* (Lea, 1856) sp.1 | Cambodia: Mekong River basin | EnsSag1 | KP795033 | KP795015 | KP795053 |
| *E.* aff. *sagittarius* (Lea, 1856) sp.2 | Thailand: Mekong River basin, artificial pond near the Ban Nong-Bua village | EnsSag2 | KX865942 | KX865813 | KX865690 |
| *E.* aff. *sagittarius* (Lea, 1856) sp.3 | Thailand: Mekong River basin, Chi River | EnsSag3 | KX865945 | KX865816 | KX865693 |
| *E.* cf. *ingallsianus* (Lea, 1852) | Laos: Mekong River basin | EnsIng | KX822641 | KX822598 | n/a |
| *E.* sp.1 | Laos: Mekong River basin | EnsSp1 | KX822642 | KX822599 | n/a |
| *Hyriopsis* sp.2 | Thailand: Mekong River basin, Chi River | HyrSp2 | KX865951 | KX865822 | KX865697 |
| *H. myersiana* (Lea, 1856) | Thailand | HyrMye | KX822645 | KX822602 | n/a |
| *H. desowitzi* Brandt, 1974 | Thailand | HyrDes | KX822644 | KX822601 | n/a |
| **UNIONINAE Rafinesque, 1820** |  |  |  |  |  |
| *Unio crassus* Philipsson, 1788 | France | UniCra | KC703878 | KC703644 | n/a |
| *U. pictorum* (Linnaeus, 1758) | Europe | UniPic | KC429109 | KC429447 | n/a |
| *U. tumidus* Philipsson, 1788 | Ukraine | UniTum | KX822672 | KX822630 | n/a |
| *Nodularia douglasiae* (Griffith & Pidgeon, 1833) | China | NodDou | KX822653 | KX822610 | n/a |
| *N. nuxpersicae* Dunker, 1848 | Vietnam | NodNux | KX822654 | KX822611 | n/a |
| *N. jourdyi* (Morlet, 1886) **comb. res.** | Vietnam | NodJou | MH248376 | MH248377 | n/a |
| *Alasmidonta marginata* Say, 1818 | USA | AlaMar | AF156502 | AF400688 | n/a |
| *Anodonta anatina* (Linnaeus, 1758) | European Russia | AnoAna | KX822632 | KX822588 | n/a |
| *Lasmigona compressa* (Lea, 1829) | USA | LasCom | AF156503 | DQ191414 | n/a |
| *Pyganodon grandis* (Say, 1829) | USA | PygGra | AF231734 | AF305384 | n/a |
| *Simpsonaias ambigua* (Say, 1825) | USA | SimAmb | KX822666 | KX822622 | n/a |
| *Strophitus undulatus* (Say, 1817) | USA | StrUnd | AF156505 | DQ191415 | n/a |
| *Anemina* sp. | Siberia | AneSp1 | KY561633 | KY561665 | KY561648 |
| *Cristaria* *plicata* (Leach, 1814) | Vietnam | CriPli | KY561634 | KY561666 | n/a |
| *Pletholophus* *tenuis* (Griffith & Pidgeon, 1833) | Vietnam | PleTen | KX822658 | KX822614 | n/a |
| *Sinanodonta* sp. | Vietnam | SinSp1 | KY561635 | KY561667 | KY561649 |
| *Lanceolaria gladiola* (Heude, 1877) | China | LanGla | KX822648 | KX822605 | n/a |
| *L. grayana* (Lea, 1834) | China | LanGra | KX822649 | KX822606 | n/a |
| **GONIDEINAE Ortmann, 1916** |  |  |  |  |  |
| *Lamprotula leaii* (Griffith & Pidgeon, 1833) | Vietnam | LamLea | KY561637 | KY561669 | KY561651 |
| *Potomida littoralis* (Cuvier, 1798) | France | PotLit | JN243905 | JN243883 | n/a |
| *Pronodularia japanensis* (Lea, 1859) | Japan | ProJap | KX822659 | KX822615 | KU946322 |
| *Gonidea angulata* (Lea, 1838) | USA | GonAng | DQ272371 | AF400691 | n/a |
| *Leguminaia wheatleyi* (Lea, 1862) | Turkey | LegWhe | KX822651 | KX822608 | n/a |
| *Microcondylaea bonellii* (A. Ferussac, 1827) | Italy | MicBon | KX822652 | KX822609 | n/a |
| **AMBLEMINAE Rafinesque, 1820** |  |  |  |  |  |
| *Amblema plicata* (Say, 1817) | USA | AmbPli | U56841 | AF305385 | n/a |
| *Actinonaias ligamentina* (Lamarck, 1819) | USA | ActLig | AF156517 | DQ191420 | n/a |
| *Lampsilis cardium* Rafinesque, 1820 | USA | LamCar | AF120653 | AF305386 | n/a |
| *Villosa iris* (Lea, 1829) | USA | VilIri | AF156524 | DQ191422 | n/a |
| *Elliptio complanata* (Lightfoot, 1786) | USA | EllCom | EU448173 | JF899181 | n/a |
| *Pleurobema sintoxia* (Rafinesque, 1820) | USA | PleSin | AF156509 | DQ191418 | n/a |
| *Quadrula quadrula* (Rafinesque, 1820) | USA | QuaQua | AF156511 | DQ191417 | n/a |
| **Out-Group Taxa** |  |  |  |  |  |
| **MARGARITIFERIDAE Haas, 1940** |  |  |  |  |  |
| *Margaritifera laosensis* (Lea, 1863) | Laos: Mekong River basin, Nam Long River | MarLao | JX497731 | KT343741 | KC845943 |
| *M. dahurica* (Middendorff, 1850) | Far East of Russia: Amur River basin, Ilistaya River | MarDah | KJ161516 | KT343747 | KJ943526 |
| *M. margaritifera* (Linnaeus, 1758) | Northwestern Russia: Onega River basin, Somba River | MarMar | KX550089 | KX550093 | KX550091 |
| *M. laevis* (Haas, 1910) | Far East of Russia: Kurile Archipelago, Kunashir Island, Sennaya River | MarLae | KJ161500 | KT343742 | KJ943523 |
| *M. middendorffi* (Rosén, 1926) | Far East of Russia: Kamchatka, Bolshaya River basin, Nachilova River | MarMid | KJ161547 | KT343745 | KJ943528 |
| **IRIDINIDAE Swainson, 1840** |  |  |  |  |  |
| *Aspatharia pfeifferiana* (Bernardi, 1860) | Zambia: Chambeshi River | AspPfe | KC429107 | n/a | KC429264 |
| *Chambardia wahlbergi* (Krauss, 1848) | Zambia: Zambezi River | ChaWah | JN243886 | JN243864 | KP184845 |
| **ETHERIIDAE Deshayes, 1832** |  |  |  |  |  |
| *Etheria elliptica* Lamarck, 1807 | Zambia: Chambeshi River | EthEll | KP184897 | KP184873 | KP184847 |
| **MYCETOPODIDAE Gray, 1840** |  |  |  |  |  |
| *Anodontites elongata* (Swainson, 1823) | Peru | AnoElo | KP184896 | KP184872 | KP184846 |
| **HYRIIDAE Swainson, 1840** |  |  |  |  |  |
| *Triplodon corrugatus* (Lamarck, 1819) | Peru | TriCor | JN243890 | JN243868 | KP184851 |
| *Castalia ambigua* Lamarck, 1819 | Peru | CasAmb | JN243889 | JN243867 | KP184848 |
| *Microdontia anodontaeformis* (Tapparone Canefri, 1883) | Guyana | MicAno | KP184909 | KP184885 | KP184861 |
| *Alathyria jacks*oni Iredale, 1934 | New Guinea | AlaJac | KP184912 | KP184888 | KP184864 |
| *A. pertexta* Iredale, 1934 | Australia: New South Wales | AlaPer | KP184910 | KP184886 | KP184862 |
| *A. profuga* (Gould, 1850) | Australia: Queensland | AlaPro | KP184913 | KP184889 | KP184865 |
| *Lortiella froggatti* Iredale, 1934 | Australia: New South Wales | LorFro | AF231746 | KP184891 | KP184867 |
| *Velesunio ambiguus* (Philippi, 1847) | Western Australia | VelAmb | KP184915 | KP184892 | KP184868 |
| **TRIGONIIDAE Lamarck, 1819** | Australia: New South Wales |  |  |  |  |
| *Neotrigonia margaritacea* (Lamarck, 1804) | Tasmania and Australia | NeoMar | U56850 | DQ279963 | DQ280034 |
| *N. lamarckii* (Gray, 1838) | Australia: Coral Sea, North Stradbroke Island, Queensland | NeoLam | KC429105 | KC429443 | KC429262 |
